# Insulin Increases Ceramide Synthesis in Skeletal Muscle

**DOI:** 10.1155/2014/765784

**Published:** 2014-05-18

**Authors:** M. E. Hansen, T. S. Tippetts, M. C. Anderson, Z. E. Holub, E. R. Moulton, A. C. Swensen, J. T. Prince, B. T. Bikman

**Affiliations:** ^1^Department of Physiology and Developmental Biology, 593 WIDB, Brigham Young University, Provo, UT 84602, USA; ^2^Department of Chemistry and Biochemistry, Brigham Young University, Provo, UT 84602, USA

## Abstract

*Aims*. The purpose of this study was to determine the effect of insulin on ceramide metabolism in skeletal muscle. *Methods*. Skeletal muscle cells were treated with insulin with or without palmitate for various time periods. Lipids (ceramides and TAG) were isolated and gene expression of multiple biosynthetic enzymes were quantified. Additionally, adult male mice received daily insulin injections for 14 days, followed by muscle ceramide analysis. *Results*. In muscle cells, insulin elicited an increase in ceramides comparable to palmitate alone. This is likely partly due to an insulin-induced increase in expression of multiple enzymes, particularly SPT2, which, when knocked down, prevented the increase in ceramides. In mice, 14 days of insulin injection resulted in increased soleus ceramides, but not TAG. However, insulin injections did significantly increase hepatic TAG compared with vehicle-injected animals. *Conclusions*. This study suggests that insulin elicits an anabolic effect on sphingolipid metabolism in skeletal muscle, resulting in increased ceramide accumulation. These findings reveal a potential mechanism of the deleterious consequences of the hyperinsulinemia that accompanies insulin resistance and suggest a possible novel therapeutic target to mitigate its effects.

## 1. Introduction

Diabetes mellitus is diagnosed on the basis of elevated blood glucose, despite significant changes in other blood markers, such as lipids, lactate, or insulin [1, 2]. The glucose emphasis is so strong that it provided the rationale for the disease name, which is understandable from a historic perspective, given that the main observable symptoms of the disease are indeed a consequence of glucose excess, including polyuria and its oft-noted sweetness [3]. However, while hyperglycemia represents the essential disorder of untreated type 1 diabetes mellitus, modern research reveals that many adverse consequences of type 2 diabetes mellitus are not a function of hyperglycemia, but rather of lipid and insulin excess [1, 4, 5].

The initial challenge to the long-embraced glucose-centric view of diabetes was issued by Denis McGarry [4] and others since [1, 6], when he posited that hyperinsulinemia and hyperlipidemia may be the more likely pathophysiological mediators of type 2 diabetes. Despite being widely prescribed in type 2 diabetes, insulin administration correlates with higher mortality rates [6, 7], and hyperinsulinemia is a factor in multiple diseases, including hypertension [8], fatty-liver disease [9, 10], PCOS [11], Alzheimer's disease [12], and more [13–15]. Though the lipid-centric aspect of McGarry's paradigm focused on triacylglycerol and free fatty acids, the sphingolipid ceramide may be the more relevant lipid regarding the deleterious consequences of type 2 diabetes and/or hyperlipidemia [16]. Indeed, increased intracellular ceramide is associated with similar diseases as hyperinsulinemia [10, 17–19].

Ceramide is an established mediator of insulin resistance in response to multiple insults [20, 21] and in diverse tissues [20, 22]. Additionally, muscle ceramide accumulation disrupts muscle metabolic function, altering mitochondrial structure, increasing ROS generation, and reducing mitochondrial respiration [23–25]. However, the role of insulin as an inducer of ceramide biosynthesis has never been explored. Not only would such a pathway identify a mechanism of negative feedback, wherein excessive insulin signaling downgrades its own signal, but also it would fit the general lipid-anabolic actions of insulin, even in skeletal muscle [26, 27]. Thus, the purpose of these studies was to explore the novel role of insulin as an inducer of ceramide biosynthesis in skeletal muscle.

## 2. Materials and Methods

### 2.1. Animals

Sixteen-week-old male C57Bl/6 mice were separated into one of two groups at 12 weeks to receive daily saline or insulin (0.75 U/kg/BW) injections for 14 days with free access to water and chow. C57Bl/6 mice are commonly used as an ideal rodent model of exploring lifestyle risks associated with diabetes and obesity that mimic human responses [28]. Studies were conducted in accordance with the principles and procedures outlined in the National Institutes of Health Guide for the Care and Use of Laboratory Animals and were approved by the IACUC (Institutional Animal Care and Use Committee) at Brigham Young University.

### 2.2. Cell Culture

C2C12 murine myoblast cells were maintained in DMEM (Dulbecco's modified Eagle's medium) plus 10% FBS (Invitrogen). For differentiation into myotubes, C2C12 myoblasts were grown to confluency and the medium was replaced with DMEM plus 10% horse serum (Invitrogen). Myotubes were used for experiments on day 4 of differentiation. For fatty acid treatment, palmitic acid (Sigma-Aldrich, catalogue number P5585) was dissolved in EtOH (ethanol) and diluted to the desired concentration in DMEM containing 2% (*w*/*v*) BSA (Sigma-Aldrich, A9576) and added to the cell culture at 0.5 mM, which is a physiological postprandial fatty acid concentration [29]. For* sptlc2* (serine palmitoyltransferase, long chain base subunit 2, also known as SPT2) knockdown, procedures were followed according to the manufacturer's instructions (Santa Cruz Biotechnology, sc-77377).

### 2.3. Lipid Isolation and Analysis

For isolation of lipids, pellets were resuspended in 900 *μ*L of ice-cold chloroform/methanol (1 : 2), incubated for 15 min on ice, and then briefly vortex-mixed. Separation of aqueous and organic phases required addition of 400 *μ*L of ice-cold water and 300 *μ*L of ice-cold chloroform. The organic phase was collected into a fresh vial, and lipids were dried in a vacuum centrifuge (Eppendorf Concentrator Plus). Lipids were characterized and quantified using a shotgun lipidomics technique on a Thermo Scientific LTQ Orbitrap XL mass spectrometer. Evaporated lipid samples were resuspended in a 2 : 1 chloroform/methanol Folch solution (200 *μ*L). The resuspended lipids were then combined with a modified 2 : 1 : 1.25 chloroform/methanol/propan-2-ol Bligh and Dyer solution (800 *μ*L) with 15 mM ammonium acetate acting as an ionizing adduct. A 1.74 *μ*M phosphatidylethanolamine internal standard (1 *μ*L) was spiked into each sample for mass calibration and characterization data alignment. Samples were analyzed using a 2.5 min mass-window-scanning method in positive-ion mode at a resolution of 60000 (fwhm (full width at half maximum) at 400* m*/*z*) for all primary MS^1^ scans. MS^2^ (tandem MS) fragmentation data were also collected and manually verified for each mass window to give additional confidence to the correct identification of abundant lipid species. Three technical replicate mass spectrometer runs were performed on each sample. Samples were injected at 10 *μ*L/min using a direct-inject ESI soft-ionization spray head from a Hamilton GASTIGHT glass syringe. The spray voltage and capillary temperature were maintained at 5.0 kV and 275°C, respectively. Each technical replicate was run in random in order to reduce systematic bias. Data were analyzed using in-house-developed peak summarization, recalibration, and lipid identification software using lipid database information from the Lipid MAPS (LIPID Metabolites and Pathways Strategy) Lipidomics Gateway database [30]. To ensure high-confidence identifications, an intensity threshold estimated to be 5% above the instrumental static signal was implemented. Lipid identities were only assigned when significantly observable peaks were identified in at least two of the three technical replicate runs. Nonzero lipid quantities were averaged from the replicate runs. The lipid species identified across different ionization states or with adducts were totaled together. Quantification was completed by normalizing total ion counts to the relative abundance of the internal standard that was spiked into each sample.

### 2.4. Protein Analysis

Tissue and cell extracts lysed and protein content was determined using a BCA protein assay (Pierce) and the sample volumes were adjusted so that precisely 50 *μ*g of protein was loaded into each lane. After the addition of sample buffer, samples were resolved by SDS/PAGE (10% gel), transferred onto nitrocellulose membranes, and immunoblotted using methods described previously [31]. After incubation with primary antibody, blots were incubated with an HRP- (horseradish peroxidase-) conjugated secondary antibody. HRP activity was assessed with ECL solution (Thermo Scientific) and exposed to film. The antibodies used were anti-SPT2 (Abcam; ab23696) and anti-rabbit IgG (Cell Signaling Technology, 7074S).

### 2.5. Real-Time qPCR

Total RNA was extracted and purified from tissues using TRIzol (Invitrogen) according to the manufacturer's recommendations. cDNA was synthesized from mRNA via reverse transcription—PCR using a commercial cDNA synthesis kit with oligo(dT) primers (iScript Select cDNA Synthesis, Bio-Rad Laboratories). Quantitative real-time PCR was performed with Evagreen Ssofast (Bio-Rad Laboratories) using a Bio-Rad Laboratories CFX Connect Real-Time PCR Detection system. Primer sequences were 5′-ACAGGATGCAGAAGGAGATTAC and 5′-CACAGAGTACTTGCGCTCAGGA as the forward and reverse primers, respectively, for* actb* (actin), 5′-TACTCAGAGACCTCCAGCTG and 5′-CACCAGGGATATGCTGTCATC for* sptlc1 *(SPT1), 5′-GGAGATGCTGAAGCGGAAC and 5′-GTATGAGCTGCTGACAGGCA for* sptlc2 *(SPT2), 5′-CACCGGTACCTCGGAGCGGA and 5′-GTTTGGGATTGATGAACAGGGGT for* des1 *(Des1), 5′-CTGTTCTACTTGGCCTGTTG and 5′-TCATGCAGGAAGAACACGAG for* lass1* (Cers1), 5′-CTCCAACGCTCACGAAATTC and 5′-ATGCAGACAGAAGATGAGTG for* lass 5* (Cers5), 5′-GTTCGGAGCATTCAACGCTG and 5′-CTGAGTCGTGAAGACAGAGG for* lass6* (Cers6), 5′-CTCGCTTGTCGTCTGCCT and 5′-TTGGCCCAGAACTCCTGTAG for FAS, and 5′-GTGCACAAGTGGTGCATCAG and 5′-CAGTGGGATCTGAGCCATCA for DGAT1. *β*-Actin reactions were performed side by side with every sample analyzed. Changes in the mRNA level of each gene for each treatment were normalized to that of the *β*-actin control mRNA according to Pfaffl [32].

## 3. Results

### 3.1. Insulin Increases Ceramide Biosynthesis in Muscle Cells

**Figure 1:**
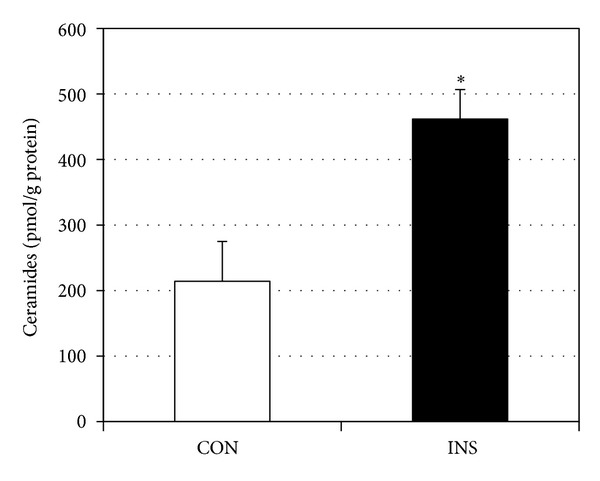
Murine myotubes were treated with vehicle (CON) or insulin (INS, 50 nM) for 16 h. Following treatment, ceramides were isolated and quantified. *P < 0.05.

**Figure 2:**
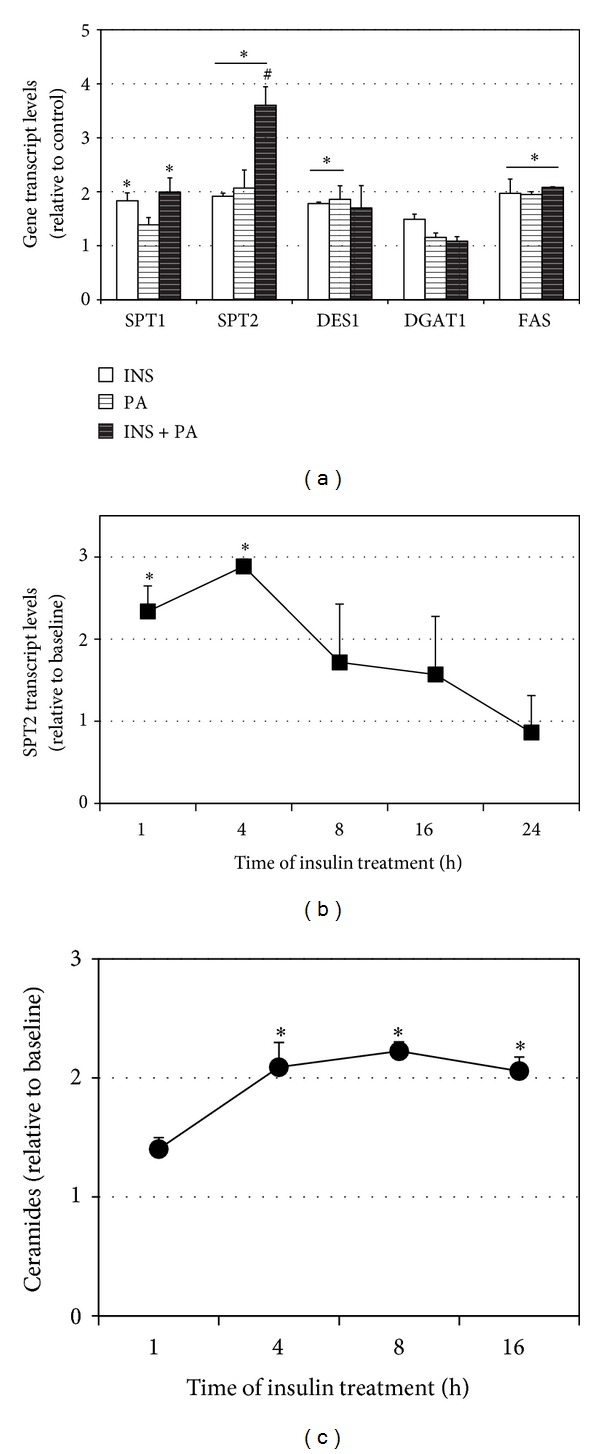
Murine myotubes were treated with insulin (50 nM) with or without palmitic acid (PA, 0.5 mM) for 16 h. (a) Following treatment, expressions of multiple enzymes involved with ceramide (SPT1, SPT2, and DES1) and TAG (DGAT1, FAS) metabolism were quantified. *P < 0.05, treatment versus control. ^#^ *P* < 0.05, INS + PA versus other treatments. (b) The time course of insulin treatment (50 nM) revealed that SPT2 gene expression was significantly increased at 1 h and peaked at 4 h. *P < 0.05, treatment versus control. (c) The time course of insulin treatment (50 nM) revealed that ceramides were significantly increased at 4 h and sustained through 16 h. *P < 0.05, treatment versus control.

The primary observation of this report is that insulin treatment increases ceramides in murine myotubes. When treated with insulin (50 nM) for 16 h, ceramide levels increased roughly twofold (Figure 1). When assessing the insulin effects on gene expression, insulin increased the expression of genes encoding ceramide biosynthetic proteins SPT1 and 2, DES1 (Figure 2(a)), and FAS, involved in triacylglycerol synthesis (Figure 2(a)). Interestingly, the effects of insulin were comparable and even greater than palmitic acid (PA) treatment [20]. Moreover, the combined effects of both insulin and PA elicited an additive response with SPT2, resulting in a roughly 75% increase. In probing SPT2 gene expression further, we found that expression was significantly increased at 1 h of insulin treatment and peaked at 4 h (Figure 2(b)). A somewhat distinct trend was observed with actual ceramides, which peaked at 8 h (Figure 2(c)) and remained comparably elevated at 16 h of treatment.

### 3.2. SPT2 Is an Important Regulator of Insulin-Induced Ceramide Accrual

**Figure 3:**
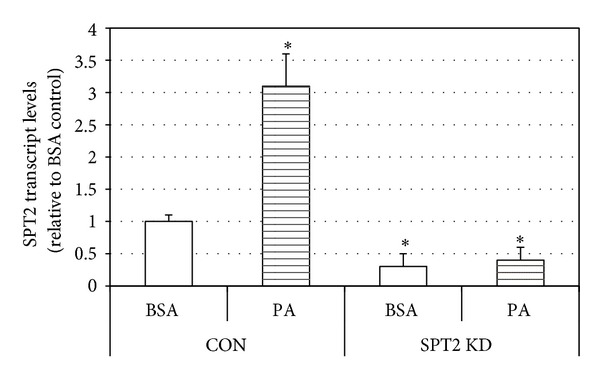
To confirm efficacy of SPT2 knockdown in murine myotubes, cells were treated with palmitic acid (PA, 0.5 mM). *P < 0.05, PA versus BSA.

**Figure 4:**
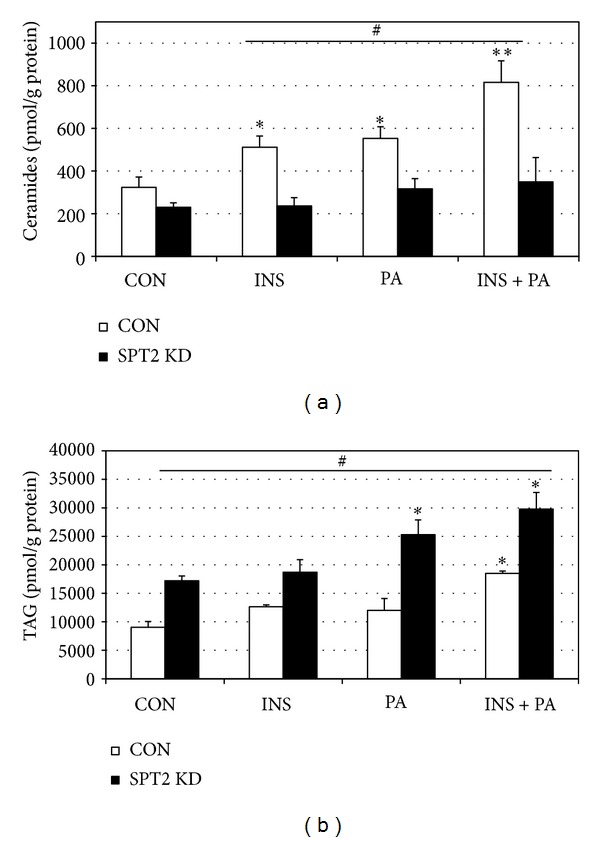
Following SPT2 knockdown, myotubes were treated with insulin (INS, 50 nM), palmitic acid (PA, 0.5 mM), or both (INS + PA). (a) Ceramides were significantly comparably elevated with INS and PA treatments, but not with SPT2 KD (*P < 0.05, treatment versus control). Both treatments together (INS + PA) resulted in an additive increase in ceramides (**P < 0.05, INS + PA versus INS and PA). SPT2 KD prevented treatment-induced increases in ceramide (^#^ *P* < 0.05, CON (scramble) versus SPT2 KD). (b) TAG levels were elevated in PA and INS + PA treatments (*P < 0.05, treatment versus control). SPT2 KD increased TAG in every condition (^#^ *P* < 0.05, SPT2 versus CON (scramble)).

SPT2 was knocked down in murine myotubes via siRNA. To confirm the functional effects of the knockdown, SPT2 transcript level was measured in control (scramble) and SPT2 siRNA-treated cells with BSA and palmitic acid (PA) incubation, which is a well-established substrate and inducer of ceramide biosynthesis [33]. Transcript levels of SPT2 were reduced compared to control cells in both BSA- and PA-treated conditions (Figure 3). Moreover, SPTKD cells failed to respond to PA treatment (Figure 3). Further, while ceramides levels were not significantly different in control and SPT2 KD cells in control conditions (CON), insulin (INS), PA, and insulin with PA (INS + PA) elicited a significant increase in ceramides in the control cells, but not the SPT2 KD cells (Figure 4(a)). Moreover, combination of insulin and PA (INS + PA) had a greater effect than INS and PA alone (Figure 4(a)). Interestingly, the SPT2 KD cells had higher TAG levels than control cells and experienced a significant increase in TAG levels with PA and INS + PA (Figure 4(b)).

### 3.3. Insulin Injections Increase Muscle Ceramides in Mice

**Figure 5:**
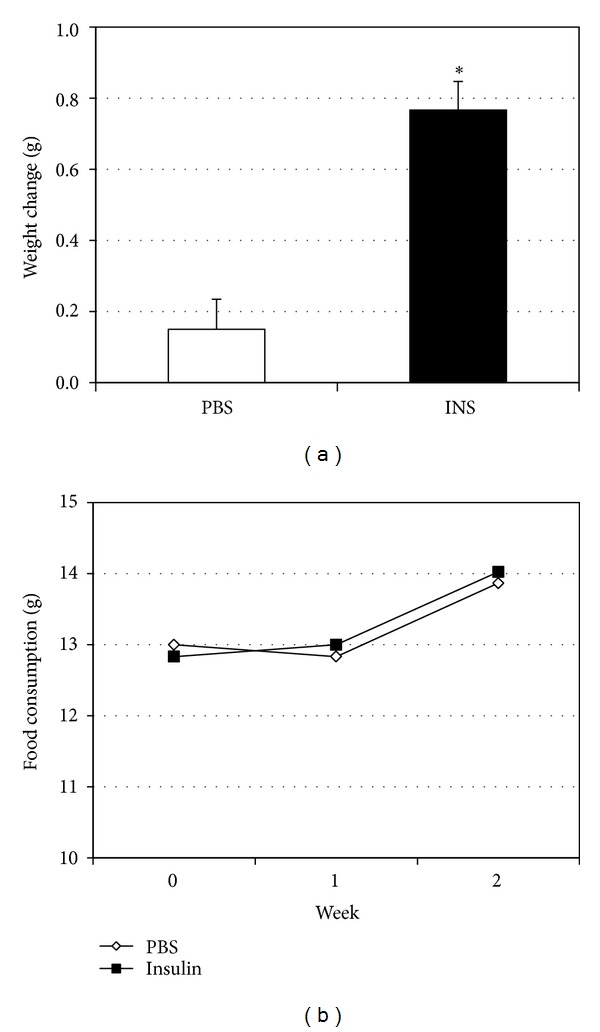
Adult male mice (12 weeks) were divided into two groups to receive insulin (INS, 50 nM) or vehicle (PBS) injections every morning for 14 d. (a) Body weights were measured before and following treatment period. (b) Weekly food consumption was determined for the week prior to injection (week 0) and for each of the two following weeks of treatment. *P < 0.05, INS versus PBS.

**Figure 6:**
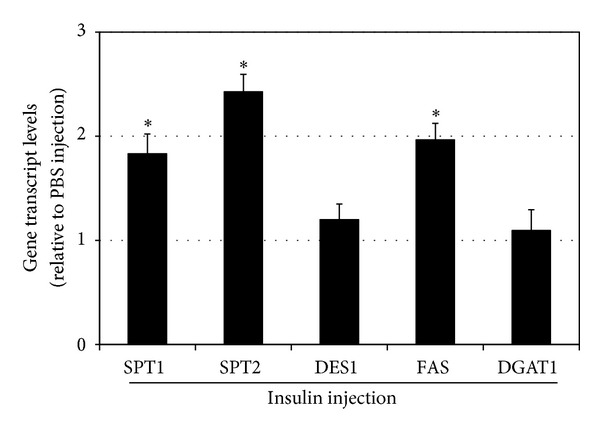
Adult male mice (12 weeks) were divided into two groups to receive insulin (INS, 50 nM) or vehicle (PBS) injections every morning for 14 d. Soleus levels of genes of multiple enzymes involved with ceramide (SPT1, SPT2, and DES1) and TAG (DGAT1 and FAS) metabolism were quantified. *P < 0.05, INS versus PBS.

**Figure 7:**
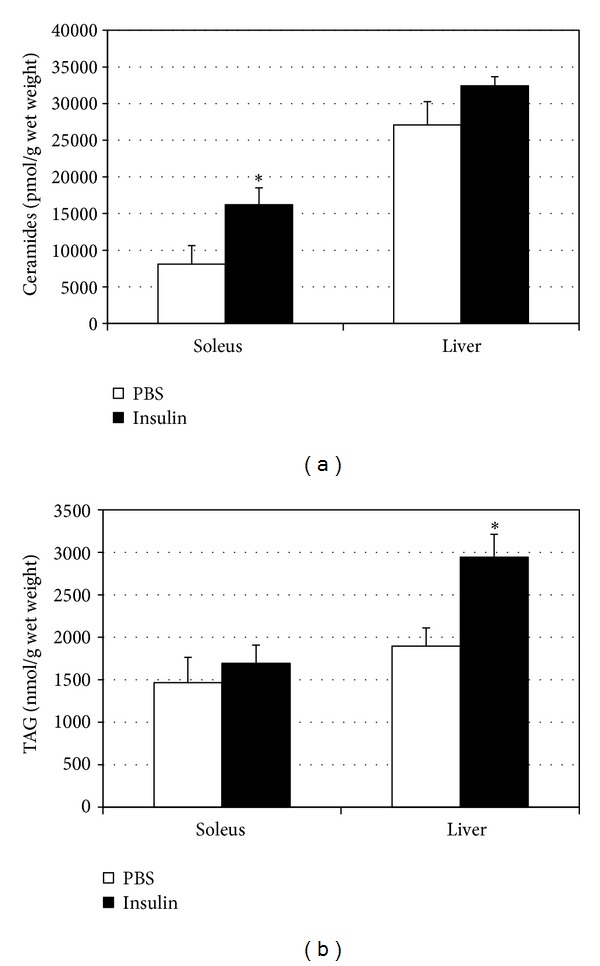
Following treatment period, soleus and liver were dissected from adult male mice receiving insulin (INS, 50 nM) or vehicle (PBS) injections every morning for 14 d. Ceramides (a) and TAG (b) were quantified from both tissues. *P < 0.05, insulin versus PBS.

Mice were injected with either insulin (0.75 U/kg) or PBS daily at the beginning of the light cycle for 14 days. Mice were allowed free access to food and water throughout the study period. At the conclusion of the study period, insulin-injected animals gained significantly greater body weight (Figure 5(a)), despite similar food consumption (Figure 5(b)). Gene expression levels in the muscle from insulin-injected animals tended to follow the trends observed in muscle cells. Specifically, levels of SPT1, SPT2, and FAS were elevated, but not DES1 and DGAT1 (Figure 6). Lastly, insulin injection had a particularly potent effect on muscle ceramide levels, but, as a comparison, not liver ceramides (Figure 7(a)). In contrast to ceramides, TAG levels in the soleus were not significantly elevated with insulin (Figure 7(b)). However, insulin injections did cause increased liver TAG (Figure 7(b)).

## 4. Discussion

This study evaluated the direct effect of insulin on skeletal muscle ceramide metabolism. The primary observation revealed that skeletal muscle cells respond to insulin treatments with a significant increase in ceramide, a finding further confirmed with increased ceramide in skeletal muscle from mice receiving daily insulin injections. This is, at least partly, a result of insulin-induced alterations in expression of enzymes involved in lipid handling (i.e., glycerolipid and sphingolipid). In particular, insulin increased expression of two isoforms of the initial and rate-limiting step in de novo ceramide biosynthesis (e.g., SPT1 and 2), as well as the final step, involving desaturation of the sphinganine backbone of dihydroceramide (DES1). Also, insulin increased FAS expression in both muscle cell cultures and whole muscle.

Our finding of increased ceramide accrual with insulin adds a layer of complexity to the abundant observations of increased skeletal muscle ceramide in insulin resistant-hyperinsulinemic states [34–36]. Whereas the basic assumption is that ceramide exacerbates insulin resistance and this is well supported [33, 37–40], the present findings suggest that ceramide accumulation may be both a cause and consequence of hyperinsulinemia. Given ceramide's actions as an insulin antagonist, it is noteworthy that insulin increases ceramide accumulation in muscle. This phenomenon may indicate a degree of insulin self-regulation, wherein insulin downregulates its signaling when in excess, such as with the hyperinsulinemia accompanying insulin-resistant states. Interestingly, recent work by Zabielski et al. [41] established that insulin deprivation increased muscle ceramides by ~50%. Specifically, after inducing type 1 diabetes mellitus through streptozotocin treatment, Zabielski et al. [41] found that insulin deprivation increased quadriceps ceramide content. Thus, combined with our results, these reports collectively suggest that both lack of and excess of insulin increase muscle ceramide accrual.

Our findings of a significant increase in TAG with SPT2 KD are relevant given the expanding appreciation of TAG accrual in muscle being benign with regard to insulin resistance. Known as the athlete's paradox, this phenomenon was first observed by Goodpaster et al. [42]. Since that time, shuttling lipid into the glycerolipid pathway in muscle is now considered a protective effect to insulin signaling. Liu et al. [43] found that mice that accumulate more TAG levels in muscle are protected against diet-induced insulin resistance. Importantly, this was associated with reduced ceramide content. We believe that a similar effect occurs with SPT2 KD; rather than shuttling carbons into sphingolipid biosynthesis with the various treatments (Figure 4), which is blunted due to knockdown of the rate-limiting step, the cell is increasing glycerolipid synthesis, evident by the increased TAG levels. Collectively, these observations suggest an insulin-sensitizing effect of ceramide inhibition and subsequent TAG synthesis.

Throughout these studies, palmitate was used as a positive control. Palmitate has long been known to increase ceramide accrual [33, 37], and it is noteworthy that insulin had a comparable effect on muscle ceramides as palmitate. Palmitate is important for ceramide biosynthesis for two reasons—as a substrate for ceramide creation (via SPT) and as an activator of ceramide biosynthetic pathways [20]. Our observation of an additive increase in ceramides with insulin and palmitate together in muscle cells is noteworthy because it reflects an environment that typifies insulin resistance—elevated insulin, increased circulating fatty acids, and increased muscle ceramides [34, 36, 44, 45]. The knowledge that both insulin and fatty acids increase ceramide highlights the potential importance of ceramide as a mediator of complications associated with elevations in both factors.

Multiple studies indicate that chronic exposure to insulin leads to insulin resistance [6], with dramatic increases in mortality from multiple diseases [46]. While the discussion is particularly relevant to type 2 diabetes, the ramifications similarly apply to type 1 diabetes. Deckert et al. [47] observed that type 1 diabetics that require less insulin have higher rates of survival. Altogether, these observations, in conjunction with our results, suggest the need for careful consideration when treating type 2 diabetes mellitus with insulin, especially when insulin levels are already elevated [6]. Whether ceramide is the primary regulator of reduced insulin sensitivity with chronic insulin is unknown.

While insulin resistance is known as a consequence of obesity [48], insulin is also critical in the expansion of adipose tissue that typifies obesity. Indeed, we observed a significant increase in body weight in the insulin-injected animals compared with the saline-injected mice (Figure 5(a)), despite no significant difference in food consumption. While the treatment period is relatively short, these findings nonetheless corroborate a substantial body of evidence showing the uniquely potent fattening effect of insulin [49–53], regardless of calories consumed [54, 55].

Insulin therapy is a common practice for treating type 2 diabetes mellitus. While this is undeniably effective at controlling blood glucose and mitigating the likelihood of hyperglycemia, the patient can suffer from the complications of hyperinsulinemia, including increased mortality [7] and weight gain [56]. Two thoughts arise from these observations. First, ideally the disease is detected in the early stage known as “prediabetes” or insulin resistance. However, this stage is typified by patients experiencing hyperinsulinemia, but not necessarily hyperglycemia. In other words, the pancreas is producing sufficient, if increased, insulin to maintain normoglycemia. Because blood glucose is typically used as the diagnostic marker of diabetes mellitus, but not insulin, many of these individuals remain undiagnosed [57]. Thus, insulin should be measured in clinical situations to allow the earlier detection of diabetes mellitus. Second, increasing efforts should be focused on lifestyle to control glucose and insulin, particularly diet. Carbohydrate-restricted diets, even in the absence of calorie restriction, are highly effective at reducing blood glucose and type 2 diabetics are often able to reduce the dose of or stop taking diabetic medications altogether [58].

## 5. Conclusions

In conclusion, our findings of increased ceramide biosynthesis with insulin provide a possible mechanism to partly explain the substantial evidence linking hyperinsulinemic conditions (e.g., insulin resistance and type 2 diabetes) to multiple disease states, especially vascular complications. For example, insulin resistance is associated with a significant increase in atherosclerosis [59] and ceramide increases atherosclerotic lesion development [60]. Similarly, insulin resistance is a common causal factor in hypertension [61], and vascular ceramide accrual compromises vasodilation [62, 63], increasing blood pressure. Thus, anticeramide therapies may prove to be a viable therapy for combating certain insulin-induced disorders, which is the focus of ongoing efforts.
